# RETRACTION: Dioscin‐loaded zein nanoparticles alleviate lipopolysaccharide‐induced acute kidney injury via the microRNA‐let 7i signalling pathways

**DOI:** 10.1049/nbt2/9767341

**Published:** 2026-03-24

**Authors:** IET Nanobiotechnology

RETRACTION: Y. Zhang, Y. Li, C. Lin, J. Zhang, H. Gao, and J. Chen, “Dioscin‐loaded zein nanoparticles alleviate lipopolysaccharide‐induced acute kidney injury via the microRNA‐let 7i signalling pathways,” *IET Nanobiotechnology*, 15, 2021: 465‐472, https://doi.org/10.1049/nbt2.12051.

The above article, published online on 12 May 2021 in Wiley Online Library (wileyonlinelibrary.com), has been retracted by agreement between the journal Editor‐in‐Chief, Duo Lin; The Institution of Engineering and Technology; and John Wiley & Sons Ltd. The retraction has been agreed due to multiple inconsistencies identified in Figure 5 [1]. The authors requested to correct the figure, however on further investigation additional concerns were noted. To summarize:

• There are repeated elements within multiple panels of Figures 5a and 5b
• There are repeated elements between the control panel of Figure 5a, and the 24 h/control panel of Figure 1 in [2] after rotation
• There are repeated elements between the DIO panel of Figure 5a, and the 24 h + 10 mg/ml panel of Figure 1 in [2] after rotation
• There are repeated elements between the DIO‐ZNP panel of Figure 5a, and the 48 h + 5 mg/ml panel of Figure 1 in [2] after rotation
• There are repeated elements between the DIO‐ZNP panel of Figure 5a, and the untreated panel of Figure 2a in [3] after rotation
• There are repeated elements between the control panel of Figure 5b, and the A549 + Green/control panel of Figure 4b in https://pmc.ncbi.nlm.nih.gov/articles/PMC6920870/ after rotation
• There are repeated elements between the LPS panel of Figure 5b, and the A549 + Green + PTX/Zein‐FA panel of Figure 4b in https://pmc.ncbi.nlm.nih.gov/articles/PMC6920870/ after rotation

After assessment of the concerns and author’s response, the editorial board have lost confidence in the results, and therefore the article is retracted.

The authors agree with the retraction.
